# Transcriptional Responses of *Bacillus cereus* towards Challenges with the Polysaccharide Chitosan

**DOI:** 10.1371/journal.pone.0024304

**Published:** 2011-09-08

**Authors:** Hilde Mellegård, Ákos T. Kovács, Toril Lindbäck, Bjørn E. Christensen, Oscar P. Kuipers, Per E. Granum

**Affiliations:** 1 Department of Food Safety and Infection Biology, Norwegian School of Veterinary Science, Oslo, Norway; 2 Molecular Genetics Group, Groningen Biomolecular Sciences and Biotechnology Institute, University of Groningen, Groningen, The Netherlands; 3 NOBIPOL, Department of Biotechnology, Norwegian University of Science and Technology, Trondheim, Norway; University of Connecticut, United States of America

## Abstract

The antibacterial activity of the polysaccharide chitosan towards different bacterial species has been extensively documented. The response mechanisms of bacteria exposed to this biopolymer and the exact molecular mechanism of action, however, have hardly been investigated. This paper reports the transcriptome profiling using DNA microarrays of the type-strain of *Bacillus cereus* (ATCC 14579) exposed to subinhibitory concentrations of two water-soluble chitosan preparations with defined chemical characteristics (molecular weight and degree of acetylation (F_A_)). The expression of 104 genes was significantly altered upon chitosan A (weight average molecular weight (M_w_) 36.0 kDa, F_A_ = 0.01) exposure and 55 genes when treated with chitosan B (M_w_ 28.4 kDa, F_A_ = 0.16). Several of these genes are involved in ion transport, especially potassium influx (BC0753-BC0756). Upregulation of a potassium transporting system coincides with previous studies showing a permeabilizing effect on bacterial cells of this polymer with subsequent loss of potassium. Quantitative PCR confirmed the upregulation of the BC0753 gene encoding the K^+^-transporting ATPase subunit A. A markerless gene replacement method was used to construct a mutant strain deficient of genes encoding an ATP-driven K^+^ transport system (Kdp) and the KdpD sensor protein. Growth of this mutant strain in potassium limiting conditions and under salt stress did not affect the growth pattern or growth yield compared to the wild-type strain. The necessity of the Kdp system for potassium acquisition in *B. cereus* is therefore questionable. Genes involved in the metabolism of arginine, proline and other cellular constituents, in addition to genes involved in the gluconeogenesis, were also significantly affected. BC2798 encoding a chitin binding protein was significantly downregulated due to chitosan exposure. This study provides insight into the response mechanisms of *B. cereus* to chitosan treatment and the significance of the Kdp system in potassium influx under challenging conditions.

## Introduction

*Bacillus cereus* is a Gram positive sporeforming bacterium and the causative agent of two forms of foodborne illness: the diarrhoeal type, where enterotoxin is produced during intestinal vegetative growth [1]–[3], and the emetic syndrome, where preformed toxin is ingested [4], [5]. Foodborne illness caused by *B. cereus* is likely to be underreported, as the symptoms are often relatively mild and normally last for less than 24 h [6]. However, cases with fatal outcome have been reported [7]–[9]. Increasing consumer request for precooked and chilled food articles today presents a larger risk of *B. cereus* food poisoning, since these are products where the competing bacterial flora has been killed due to different treatment processes, which allow the surviving bacterial spores to grow to levels able to cause disease under favorable conditions [10].

Various food preservative techniques, such as heat treatment, temperature reduction and the addition of substances like nitrite, weak organic acids and bacteriocins to food articles, are employed to reduce the risk of foodborne illness. An increasing tendency among consumers to prefer products supplemented with naturally occurring rather than industrial additives [11], stimulates a search for novel preservatives of natural origin. The polysaccharide chitosan is biodegradable and possesses relatively low cytotoxicity towards mammalian cells [12]–[14] and exhibits potential applications in food preservation [15], [16]. Inhibitory activity against spoilage yeast and bacteria, including pathogens like *B. cereus*, has been described [17]–[22].

Commercial production of chitosan is usually obtained by partial de-*N*-acetylation of chitin, the major structural component of the exoskeleton of crustaceans [23]. The degree of *N*-acetylation (F_A_) and the molecular weight (MW) are chitosan characteristics shown to be important as determinants of antibacterial activity [22], [24]–[26]. According to general acid-base theory, a majority of the amino groups of the glucosamine units of the biopolymer will be positively charged belowthe pK_a_-value of chitosan, which is reported to be 6.2–7.0, depending on the chitosans applied and the test conditions [27]–[30]. This polycationic nature of chitosan enables it to bind to negatively charged surfaces, such as polymers, tissues, cells and DNA, through both electrostatic and non-electrostatic interactions [31]–[33], which is believed to constitute the basis of its antimicrobial activity. It is therefore essential that experiments involving chitosan are conducted at a pH below the pK_a_. Permeabilization of Gram positive and Gram negative bacterial cells upon chitosan treatment with the subsequent release of intracellular compounds, such as K^+^ ions and nucleotides, are described by several authors [22], [34]–[37]. Because of the osmotic gradient it is likely that cell water will follow in hyperosmotic environments, causing a reduction of cell membrane turgor and dehydration of the cells.

There are also other proposed mechanisms of action of chitosan, including blockage of nutrient flow by forming a polymer layer around bacterial cells [38]. In some reports, including a review by Rabea et al. [39], it is also suggested that chitosan might appear intracellularly and bind to DNA and thereby interact with mRNA and protein synthesis, but plausible experimental evidence has not yet been obtained. This model is also questioned by Raafat et al. [34], [35], who point out that chitosan would then have to pass through both the cell wall and the cytoplasmic membrane to reach its target. Cell wall and/or membrane permeabilization is therefore more likely to contribute to the mechanism of action of chitosan. Even though there is a substantial amount of publications describing the antibacterial activity of chitosan, including perturbation of bacterial membranes, the exact molecular mechanism of action and responses of treated bacterial cells are not well documented. In the before-mentioned study by Raafat et al. [34], [35], transcriptome analysis of *Staphylococcus aureus* cells exposed to chitosan (weight average molecular weight (M_w_) of approximately 240 kDa, F_A_ = 0.13) showed that expression of genes involved in stress and autolysis regulation, in addition to expression of genes associated with energy metabolism and growth, were significantly and more than twofold altered. The authors suggested that the mechanism of action of chitosan is related to the occurrence of multiple events rather than chitosan targeting one single molecular system. Binding to and immobilization of lipoteichoic acids of Gram positive bacterial cell walls by chitosan with possible cytoplasmic membrane destabilization, were hypothesized to be part of the chitosan mechanisms.

To our knowledge, there are no other studies performed on the transcriptional response of bacteria to chitosan. Thus, we have investigated the response of *B. cereus* to chitosan by conducting DNA microarray experiments. Genes constituting the potassium uptake system Kdp were significantly upregulated when *B. cereus* ATCC 14579 (hereafter denoted *B. cereus* 14579) was exposed to two chitosans of similar M_w_ but different F_A_. This coincides well with published reports on bacterial cell permeabilization and subsequent loss of potassium upon chitosan exposure. Phenotypic behavior of the *kdp* mutant when challenged with mild to pronounced NaCl mediated osmotic shock and growth in potassium limiting medium was characterized and compared with the wild-type strain, in addition to susceptibility testing towards chitosan A and B for both strains. Information on regulation of potassium acquisition is limited in Gram positives, and possible explanations to the observed behavior of the Kdp system deletion mutant are discussed here.

## Results

### Characterization of chitosans

Calculated characteristics of the chitosans obtained by size-exclusion chromatography with on-line multi-angle laser light scattering (SEC-MALLS), in addition to F_A_ of the chitosans, are given in Table 1. Note that in the following we will refer to the two different chitosans by names A or B from this table. Details on SEC behavior of some chitosans with acetylation of 0.16 are given in Mellegård et al. [22]. Chitosan with F_A_ = 0.01 showed a similar SEC trend as for the F_A_ = 0.16 chitosans (data not shown). Chitosans are inherently polydisperse in MW, a feature that also persists through the random degradation by nitric acid. Hence, the polydispersity index, defined as M_w_/M_n_, is typically close to 2.

**Table 1:** Characteristics of the chitosan samples included in the study.^a^

Chitosan	M_w_ (kDa)	M_n_ (kDa)	M_w_/M_n_	DP_n_ (calculated)	F_A_
A	36.0	18.0	2.0	81	0.01
B	28.4	17.0	1.7	85	0.16

a Abbreviations: M_w_, weight-average molecular weight; M_n_, number-average molecular weight; DP_n_, number-average degree of polymerization; F_A_, degree of acetylation.

### Transcriptional responses to chitosan exposure

In this study we have performed DNA microarray analysis to gain insight into the transcriptional responses of *B. cereus* 14579 exposed for 30 min to subinhibitory concentrations (50 µg/mL) of two chitosans differing in macromolecular characteristics (Table 1), which have been shown to be among the most active chitosan preparations included in earlier studies [22], [40]. CyberT analysis showed a significantly altered expression of 104 genes upon chitosan A exposure and 55 genes when treated with chitosan B (Bayesian P≤1.0×10^−4^, cut-off value ≥2). A complete list of genes significantly affected by chitosan A and B is presented in Tables S1 and S2, respectively, while in Tables 2, 3, 4, 5 significantly differentially expressed genes with cut-off value ≥3 are shown for the same chitosans. SMART searches [41] were performed to detect different protein domains of the annotated genes.

**Table 2:** Summary of upregulated genes (Bayesian P≤1.0×10^−4^, cut-off value ≥3) in *B. cereus* 14579 upon 50 µg/mL chitosan A treatment.

locus tag	Expression ratio^a^	Significance (p-value)^b^	annotation^c^	feature^d^
Upregulated				
**BC3719**	8.7	10^−7^	1-phosphofructokinase	phosphomethylpyrimidine kinase domain
**BC0755**	6.8	10^−5^	potassium-transporting ATPase subunit C	SS, TMS(1)
**BC1043**	6.7	10^−5^	peptidylprolyl isomerase	SS, rotamase domain
**BC2609**	5.9	10^−5^	cytochrome P450	p450 domain
**BC3720**	5.8	10^−6^	DeoR family transcriptional regulator	HTH
**BC4016**	5.7	10^−6^	cyclodextrin transport ATP-binding protein	AAA, transport-associated OB domain
**BC3718**	5.1	10^−5^	PTS system, fructose-specific II ABC component	phosphotransferase system domains
**BC0753**	5.1	10^−4^	potassium-transporting ATPase subunit A	TMS(10)
**BC2603**	4.4	10^−4^	hypothetical protein	SS, TMS(5)
**BC4366**	4.3	10^−5^	cystathionine beta-lyase	
**BC4015**	4.1	10^−5^	oligo-1,6-glucosidase	amylase domain
**BC0754**	3.7	10^−5^	potassium-transporting ATPase subunit B	TMS(3), AAA, hydrolase
**BC4062**	3.7	10^−5^	hypothetical protein	SS, CD
**BC3515**	3.6	10^−4^	glycosyltransferase	
**BC4761**	3.6	10^−4^	methionine adenosyltransferase	S-adenosylmethionine synthetase domains
**BC5448**	3.6	10^−4^	UDP-glucose 4-epimerase	epimerase
**BC3466**	3.5	10^−5^	ferrichrome-binding protein	SS, PPD
**BC1461**	3.5	10^−5^	DNA integration/recombination/invertion protein	integrase domain
**BC4242**	3.5	10^−5^	H^+^/Na^+^-glutamate symport protein	SS, Na^+^:dicarboxylate symporter domain
**BC4802**	3.4	10^−5^	hypothetical protein	SS
**BC5387**	3.4	10^−4^	phosphotransacetylase	
**BC0413**	3.4	10^−5^	exo-α-1,4-glucosidase	amylase domain
**BC5380**	3.3	10^−4^	ferrichrome-binding protein	SS, PPD
**BC3423**	3.3	10^−5^	ArsR family transcriptional regulator	HTH
**BC1528**	3.2	10^−5^	hypothetical protein	TMS(4), peptidase
**BC3523**	3.1	10^−5^	hemolysin II	leukocidin domain
**BC2969**	3.0	10^−4^	hypothetical protein	monooxygenase domain

a The ratio of gene expression is shown. Ratio: expression in chitosan treated samples over that in untreated samples.

b Bayesian *p* value.

c Putative function of protein as annotated in the *B. cereus* ATCC14579 genome sequence.

d Domains detected using SMART search (http://smart.embl-heidelberg.de/) [40]. SS, signal sequence; TMS(n), transmembrane segment (n is the number of such domain); CD, conserved domain of unknown function; PPD, periplasmic domain; HTH, helix turn helix,; FtsX, FtsX like permease family; AAA, ATPase domain.

**Table 3:** Summary of downregulated genes (Bayesian P≤1.0×10^−4^, cut-off value ≥3) in *B. cereus* 14579 upon 50 µg/mL chitosan A treatment.

locus tag	Expression ratio^a^	Significance(p-value)^b^	annotation^c^	feature^d^
Downregulated				
**BC2134**	0.3	10^−4^	bifunctional uroporphyrinogen-III	methylase domain
**BC0744**	0.3	10^−5^	hydroxymethylpyrimidine transport system permease protein	SS, TMS(6)
**BC4927**	0.3	10^−5^	cell surface protein	TMS(2)
**BC3855**	0.3	10^−4^	putative alkaline-shock protein	
**BC2121**	0.3	10^−5^	respiratory nitrate reductase γ chain	nitrate reductase domain
**BC3223**	0.3	10^−6^	ABC transporter permease protein	SS, FtsX
**BC0492**	0.3	10^−5^	pyruvate formate-lyase activating enzyme	radical SAM domain
**BC0402**	0.3	10^−5^	cystine-binding protein	SS, bacterial periplasmic substrate-binding proteins
**BC3651**	0.3	10^−6^	urocanate hydratase	urocanase
**BC0403**	0.3	10^−5^	glutamine transport ATP-binding protein glnQ	AAA
**BC2778**	0.3	10^−4^	acetoin dehydrogenase E1 component β-subunit	transketolase
**BC0404**	0.3	10^−5^	methyl-accepting chemotaxis protein	SS, TMS(2), histidine kinases/adenylyl cyclases/methyl binding proteins/phosphatases domain
**BC2132**	0.3	10^−6^	precorrin-2 dehydrogenase	
**BC4793**	0.3	10^−5^	cytochrome d ubiquinol oxidase, subunit II	cytochrome oxidase domain
**BC2798**	0.2	10^−4^	chitin binding protein	chitin binding domain, carbohydrate-binding domain
**BC2133**	0.2	10^−6^	CbiX protein	CbiX domains
**BC2136**	0.2	10^−6^	nitrite reductase [NAD(P)H] large subunit	oxidoreducate, ferredoxin domain
**BC0503**	0.2	10^−5^	hypothetical protein	SS, CD, TMS(2)
**BC2779**	0.2	10^−4^	acetoin dehydrogenase E1 component α-subunit	dehydrogenase
**BC0412**	0.2	10^−4^	FAD-dependent oxidase	FAD-binding domain
**BC3650**	0.2	10^−6^	imidazolonepropionase	amidohydrolase
**BC2776**	0.2	10^−7^	dihydrolipoamide dehydrogenase	oxidoreductasedomain
**BC2777**	0.2	10^−7^	branched-chain alpha-keto acid dehydrogenase subunit E2	biotin attachment domain, dehydrogenase domain
**BC0406**	0.1	10^−6^	arginine deiminase	aminidotransferase
**BC0407**	0.1	10^−7^	ornithine carbamoyltransferase	carbamoyl-P binding domain; Asp/Orn binding domain
**BC0409**	0.1	10^−7^	carbamate kinase	kinase
**BC2992**	<0.1	10^−9^	ribosomal-protein-alanine acetyltransferase	acetyltransferase
**BC0408**	<0.1	10^−8^	arginine/ornithine antiporter	permease; TMS(1)

See Table 2 for explanatory footnotes.

**Table 4:** Summary of upregulated genes (Bayesian P≤1.0×10^−4^, cut-off value ≥3) in *B. cereus* 14579 upon 50 µg/mL chitosan B treatment.

locus tag	Expression ratio^a^	Significance (p-value)^b^	annotation^c^	feature^d^
Upregulated				
**BC0753**	7.5	10^−8^	Potassium-transporting ATPase A chain	TMS(10)
**BC0754**	6.9	10^−7^	Potassium-transporting ATPase B chain	TMS(3), AAA, hydrolase
**BC0755**	6.4	10^−9^	Potassium-transporting ATPase C chain	SS, TMS(1)
**BC0814**	4.3	10^−5^	ABC transporter permease protein	TMS(1), FtsX
**BC1739**	3.5	10^−4^	H^+^/Na^+^-glutamate symport protein	TMS(9)
**BC1461**	3.3	10^−5^	DNA integration/recombination/invertion protein	integrase
**BC4813**	3.3	10^−5^	hypothetical protein	
**BC0756**	3.3	10^−6^	sensor protein (KdpD)	universal stress protein domain
**BC3738**	3.2	10^−5^	Iron(III) dicitrate-binding protein	SS, PPD
**BC1612**	3.1	10^−6^	Na^+^/H^+^ antiporter NapA (inosine-dependent germination)	TMS(11)
**BC3093**	3.1	10^−4^	aspartate ammonia-lyase	lyase, fumarase
**BC5448**	3.0	10^−4^	UDP-glucose 4-epimerase	epimerase
**BC0816**	3.0	10^−5^	periplasmic component of efflux system	SS, superfamily of outer membrane efflux proteins

See Table 2 for explanatory footnotes.

**Table 5:** Summary of downregulated genes (Bayesian P≤1.0×10^−4^, cut-off value ≥3) in *B. cereus* 14579 upon 50 µg/mL chitosan B treatment.

locus tag	Expression ratio^a^	Significance (p-value)^b^	annotation^c^	feature^d^
Downregulated				
**BC2798**	0.2	10^−5^	chitin binding protein	chitin binding domain, carbohydrate-binding domain

See Table 2 for explanatory footnotes.

**Figure 1:**
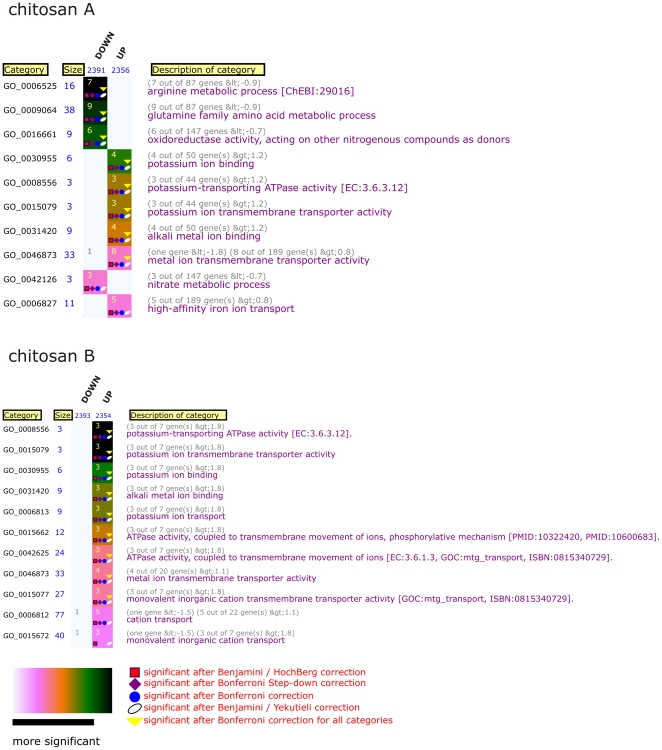
FIVA analysis of differentially regulated genes in *B. cereus* 14579 cells exposed to chitosan. Graphical representation of the over-represented categories in the transcriptome analysis of chitosan A (above) and chitosan B (below) treated *B. cereus* cultures using FIVA software [41]. The size of each cluster is displayed next to the cluster name. Numbers in each rectangle represent absolute values of occurrences. The significance of occurrences is visualized in a colour gradient which is displayed at the bottom of the figure. The description of each category is placed at the right. Multiple testing correction results are visualized using five different symbols to distinguish between the individual corrections. The number of symbols placed in each rectangle corresponds to the number of multiple testing corrections after which the annotation is found significant.

According to FIVA (Functional Information Viewer and Analyzer) analysis [42], genes involved in ion transport, especially transport of potassium, were found significantly upregulated upon exposure to both chitosans (Figure 1). BC0753-BC0756 encode the homologues of *E. coli* Kdp ATPase A–C chain in addition to the sensor protein KdpD, and these genes were all increased in expression more than twofold upon chitosan B treatment. Exposure to chitosan A stimulated the expression of genes encoding the Kdp ATPase A–C chain, but the KdpD protein was not significantly upregulated. Genes constituting the Kdp ATPase system encode a high-affinity K^+^ specific influx system, which is common among both Gram negative and Gram positive bacteria, according to BLAST searches performed with Kdp protein sequences [43].

Genes encoding several hypothetical proteins were also significantly affected upon chitosan treatment. The expression of genes involved in arginine and proline metabolism was downregulated with both chitosans. Also, for chitosan A, expression of genes involved in nitrogen, alanine, aspartate and pyruvate metabolism, in addition to the gluconeogenesis, were decreased. BC2798 encoding a chitin binding protein was significantly downregulated upon treatment of the test strain with both chitosans.

The expression of the BC0753 gene was followed using quantitative real time RT-PCR (qPCR) to validate our microarray results and verify whether upregulation of BC0753 is specific to chitosan treatment. qPCR showed 7.3±1.6 and 20.2±0.5 folds upregulation of BC0753 in samples treated with chitosan A and B, respectively. The expression level of BC0753 was not significantly changed in *B. cereus* 14579 samples treated with the bacteriocin nisin (1.1±0.1), slightly upregulated in the presence of the bacteriocin bacitracin (2.2±0.5) and downregulated in the presence of enterocin AS-48 (0.3±0.05).

### Characterization of growth of the BC0753-BC0756 deletion mutant under different conditions

As the BC0753-BC0755 genes were significantly upregulated in response to treatment with both chitosan A and B, in addition to the BC0756 gene in response to chitosan B, the genes BC753-BC0756 (encoding proteins involved in K^+^ uptake) were deleted from the chromosome of *B. cereus* 14579 as described in the Materials and Methods section. Growth curves recorded as optical density at 600 nm (OD_600_) measurements in Iso-Sensitest Broth (Iso-SB) with 100 mM 4-Morpholineethanesulfonic acid (MES) pH 6.0 at 37°C for 9 h did not differ noticeably between the wild-type and the mutant strain (data not shown) and yielded 5×10^8^–2×10^9^ CFU/mL at the end of the experiments for both strains. According to Epstein [44], the need for K^+^ under physiological growth conditions is rather low and the full capacity of the transport system is therefore not fully acknowledged under such conditions, which may also have implications for our results.

Growth in a modified Spizizen's minimal medium (SMM) [45], with sodium salts replacing the potassium salts and a supplementation of 1.0 mg/mL arginine and trace elements, in addition to different concentrations of KCl (0, 1, 2 or 3 mM), were compared for the *B. cereus* 14579 wild-type strain and *kdp* mutant strain. No major differences in growth yield were observed, as both strains gave 10^7^ CFU/mL after 18 h at 37°C in the minimal medium where no KCl was added and 10^7^–10^8^ CFU/mL where the medium was supplemented with 1–3 mM KCl.

**Figure 2:**
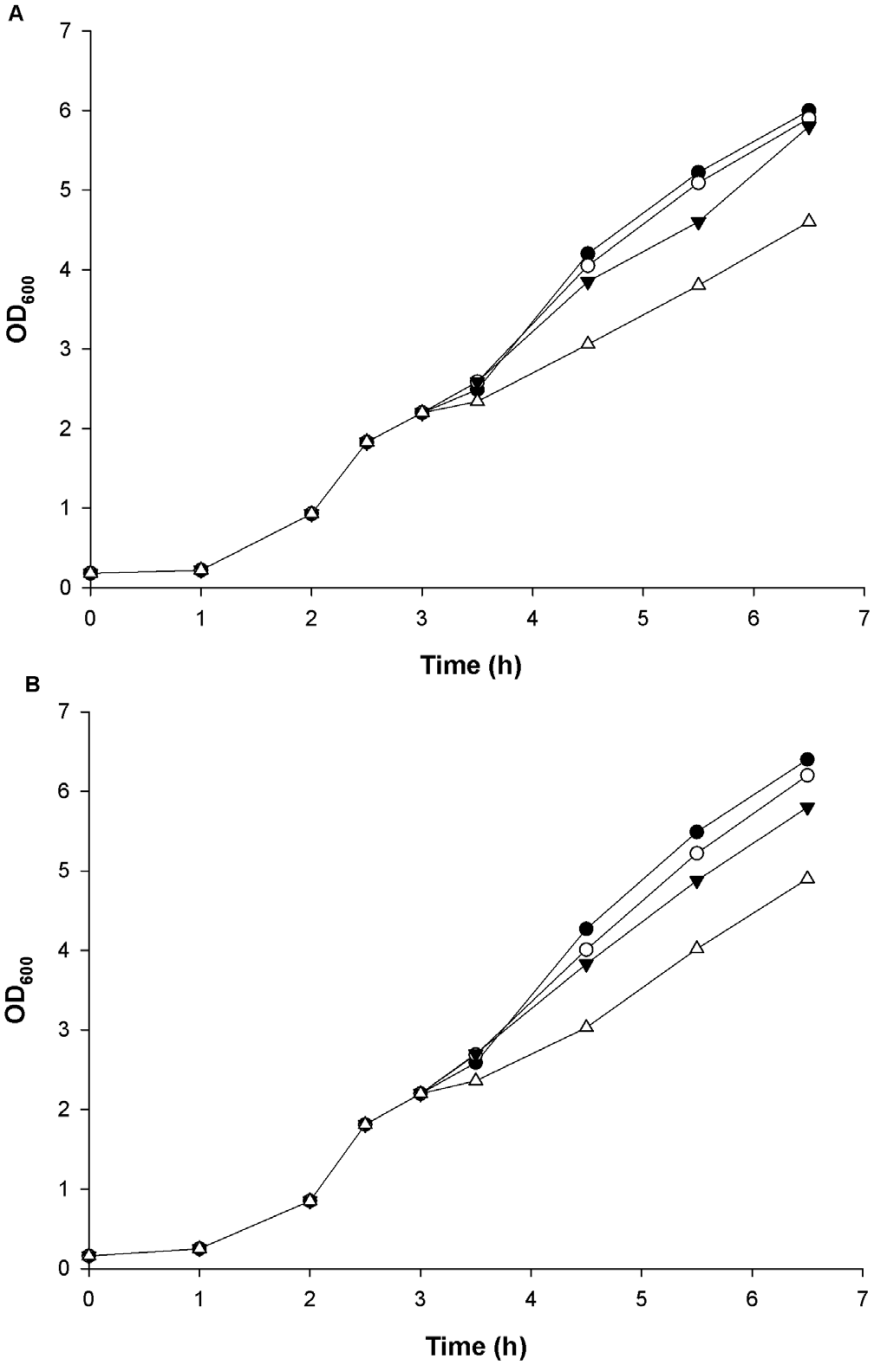
Growth patterns of *B. cereus* 14579 wild-type and mutant strain subjected to osmotic stress. Representative recordings of growth (OD_600_ measurements) of the two test strains in Iso-SB 100 mM MES at pH 6.0 subjected to osmotic upshift mediated by different concentrations of NaCl. Wild-type strain (A) and *kdp* mutant strain (B) of *B. cereus* 14579 exposed to 0 M (•), 0.25 M (○), 0.5 M (▾) and 1.0 M (Δ) NaCl.

The effect of salt stress on growth of *B. cereus* 14579 was assessed for the wild-type and the mutant strain. Both strains displayed little growth reduction when challenged with 0.25 M NaCl in Iso-SB 100 mM MES at pH 6.0. Increasing concentrations of NaCl up to 1.0 M decreased the growth rate in a concentration-dependent manner, reflected as OD_600_ measurements, but there were no observable differences in growth pattern between the two test strains. Representative recordings of growth of the test strains in 0, 0.25, 0.5 and 1.0 M NaCl are shown in Figure 2.

### Chitosan susceptibility assay

The minimum inhibitory concentrations (MIC) of chitosan A and B towards *B. cereus* 14579 wild-type and the mutant strain lacking *kdp* genes are given in Table 6 and did not differ significantly (Student's *t*-test). The corresponding minimum bactericidal concentrations (MBC) of the two chitosans towards the mutant strain were slightly higher compared to the wild-type, but did not differ more than one twofold dilution unit.

**Table 6:** Antimicrobial susceptibility to chitosan samples A and B.^a^

Chitosan	*B. cereus* ATCC 14579		*B. cereus* ATCC 14579Δ*kdp*	
	MIC (mg/ml)	MBC (mg/ml)	MIC (mg/ml)	MBC (mg/ml)
A	0.063±0.0	0.063±0.0	0.084±0.02	0.125±0.0
B	0.084±0.02	0.084±0.02	0.084±0.02	0.145±0.06

a Averages of minimum three separate experiments ± standard errors.

## Discussion

In this study, we have provided insight into the response mechanisms of *B. cereus* challenged with subinhibitory chitosan concentrations. Based on the results from DNA microarray experiments, where genes encoding a potassium influx system (the Kdp system) were significantly upregulated, we constructed a mutant lacking the various *kdp* genes. The mutant strain did not exhibit increased susceptibility to chitosan A or B compared to the wild-type strain, reflected as MIC/MBC values. Also, no growth differences were observed upon exposure to salt stress or potassium limiting conditions.

Potassium is the dominant monovalent cation in bacterial cells and has important functions as an osmotic solute, enzyme activator, internal pH regulator and a second messenger, the latter by enhancing accumulation of compatible solutes, such as trehalose, proline, glycine and betaine. Therefore, the intracellular K^+^ concentration is tightly regulated [44], [46], [47]. Potassium transport is well documented in the Gram negative *E. coli* [44], [48], but there is limited information on this subject in Gram positives. There are at least three major K^+^ influx transport systems described in *E. coli*. Two of these systems are constitutively expressed (Trk and Kup) and maintain K^+^ requirements by the cells under normal physiological conditions, while the Kdp system is regulated at the level of expression and only comes into play when the bacterial cells are subjected to osmotic upshift or when the other uptake systems do not provide enough potassium for the requirements of the cells [44], [49]. The Kdp system is also described as having potential to compensate for defects in other potassium transporters (Trk) in *E. coli* [50]. In *E. coli*, the Kdp ATPase complex consists of the KdpA, KdpB, KdpC and KdpF subunits and is encoded for by the *kdpFABC* operon [44]. The sensor kinase KdpD and the response regulator KdpE modulate the expression of the *kdpFABC* genes in response to cellular stimuli [49], [51]. Until recently, KdpD was hypothesized to react to low cell turgor (tension across the cell envelope), influenced by the intracellular K^+^ concentration [44]. However, a more recent study showed that turgor reduction is probably not the stimulus for KdpD activity in *E. coli* [52], and in a review paper by Heermann and Jung [51], KdpD was suggested to respond to three different cytoplasmic stimuli, namely potassium concentrations, ionic strength and level of ATP. Although the Kdp ATPase and the KdpD/KdpE systems are described to be common among different bacterial species [43], [51], the most prominent low G + C Gram positive model bacterium *B. subtilis*, unlike *B. cereus*, does not possess a Kdp system, as complete genome sequencing of *B. subtilis* has not revealed any *kdp*-related genes [53]. According to Stumpe et al. [49], the Kdp system may not be essential to prokaryotic organisms in general, since *kdp* genes are also absent from the genomes of other bacteria, such as *Haemophilus influenzae*, *Vibrio alginolyticus* and *Enterococcus hirae* and not described for any archae bacteria.

Concerning other major bacterial K^+^ regulating systems, the high rate, low affinity K^+^ influx complex Trk is regarded as very common in prokaryotes, as similar systems to Trk in *E. coli* has been found in most organisms where this have been studied, including the rather small genome (1.8 Mbp) of *H. influenzae* [49], [54]. Together with the Kdp complex, the Trk system is denoted as a mediator in potassium influx when *E. coli* cells are exposed to salt stress [49]. Another potassium transporting complex, Kup, is reported not to be involved in the adaptation process of *E. coli* cells to osmotic challenges [55]. However, the Kup system is of physiological importance in K^+^ uptake by *E. coli*, as partial deletion of the *kup* encoding gene led to lower K^+^ uptake in a study by Schleyer and Bakker [56].

Studies on potassium specific uptake systems in the Gram positive model organism *Bacillus subtilis* are limited, and potassium acquisition in *B. cereus* is even less characterized. In *B. subtilis*, a homologue to one of the Trk subunits in *E. coli* is described and shown to be involved in K^+^ uptake, but the amino acid identity is rather low [57]. A tetracycline-metal/H^+^ antiporter with additional monovalent cation/H^+^ activity showing a net K^+^ uptake (TetA(L)) is described to possess a physiological K^+^ uptake in *B. subtilis*, as TetA(L) deletion strains displayed reduced growth in low K^+^ media [58]. Subsequent studies have also revealed the existence of another K^+^ transporting system in *B. subtilis*. Holtmann et al. (2003) [59] described two novel major potassium uptake system present in *B. subtilis*, named KtrAB and KtrCD, which are homologues to KtrA and KtrB in *V. alginolyticus*. Homologues to KtrA and KtrB have been described for several bacterial species [60], including *B. cereus* E33L and *B. cereus* Q1, where KtrA is annotated as a Trk family potassium uptake protein [61], [62].

Through earlier studies we have demonstrated the permeabilizing effect of chitosan B (Table 1) on *B. cereus* 14579 cells, reflected as a leakage of intracellular potassium [22], and the same membrane perturbing effect of chitosan has also been observed by other workers with different bacterial species [34]–[37]. Our transcriptomic data revealed significant upregulation of *kdp* genes in *B. cereus* 14579 (BC0753-BC0756) in response to chitosan treatment, which further strengthens these results and thereby the prevailing theory on mode of action of chitosan. In this study, a mutant absent of BC0753-BC0756 (encoding the Kdp system/KdpD) did not display increased sensitivity to chitosan in the assays applied compared to the wild-type. This may originate from different reasons. As mentioned above, the potassium regulation of *Bacillus* spp. is not very well described, which means that there is a possibility of K^+^ regulating systems similar to that described for *E. coli* or even unknown K^+^ uptake systems acting on potassium loss and thereby superseding the Kdp ATPase complex. The Kdp system may therefore not be essential for survival and growth under hyperosmotic or potassium limiting conditions in *B. cereus*. Also, the effect of deleting the Kdp system might not be prominent enough on K^+^ uptake to be reflected in decreased growth rate in our assays. Atomic adsorption and flame photometry are examples of other methods that have been applied to study K^+^ influx in bacterial cells [59], [60], in addition to OD measurements. A third explanation might be the presence of contaminating K^+^ in our minimal medium (SMM), as some of the chemicals added to this medium are reported to potentially contain very low amounts of K^+^ (calculated to a maximum of 7 µM K^+^ in total) (see Materials and Methods section). Low amounts of K^+^ in the growth medium might influence on the Kdp system, as an older study on cation transport in *E. coli* using radioactive K^+^ (K^42^) showed that the K^+^ exchange flux of this system was in fact reduced by more than threefold when the external concentration of K^+^ was elevated from 30 µM to 1 mM [63]. Kdp inhibition by K^+^ in the test medium may to some extent explain the similar growth patterns of the two test strains in the osmotic upshift assay and MIC assay, since the K^+^ content in Iso-SB 100 mM MES was measured to 2.6 mM. The Kdp system may therefore not be functional even in the wild-type strain in the mentioned assays, if the findings by Rhoads and Epstein [63] also apply for this K^+^ uptake system in *B. cereus*.

In the before-mentioned study on chitosan and gene regulation in *S. aureus* [34], [35], the level of transcription of *kdpA* and *kdpC*, encoding subunits of the Kdp system, were determined to be approximately 1.5 fold higher in chitosan treated cells compared to untreated cells. However, significant upregulation of genes encoding the KdpB subunit and the sensor and regulatory system KdpD/KdpE was not detected. These findings were not discussed in the paper (threshold value of significantly differentially expressed genes displayed in the main body of the paper was set to ≥2.0). The expression profiles of *B. cereus* 14579 upon treatment with chitosan A and B showing significant and more than 3.7 fold upregulation of the genes encoding the Kdp system, do not coincide with published profiles of other bacterial inhibitors, such as disinfectants, bacteriocins (AS-48) or acidulants. Ceragioli et al. [64] compared transcriptomic analysis of *B. cereus* 14579 subjected to the disinfectants benzalkonium chloride (BC), sodium hypochlorite, hydrogen peroxide and peracetic acid. The data revealed general and oxidative stress responses upon treatment with all test substances, in addition to disinfectant specific responses. As for chitosan, BC is thought to act as a bacterial membrane-active agent leading to leakage of intracellular material. Genes involved in fatty acid metabolism were upregulated upon BC exposure in the mentioned study, and no significantly altered expression of genes involved in the Kdp complex was detected with any of the four test agents. Upon treatment with enterocin AS-48, which is a cyclic peptide produced by *Enterococcus faecalis*, genes encoding membrane associated or periplasmic proteins were upregulated in the type-strain of *B. cereus*, while genes involved in arginine and ornithine catabolism were significantly downregulated [65]. The cytoplasmic membrane is also described as the prime target for AS-48, but this bacteriocin acts through opening up pores and disturbing the proton motive force, like cationic antibacterial peptides in general [66], instead of membrane permeabilization. Also, the gene expression pattern of acid-stressed *B. cereus* strains, including the type-strain, exposed to different acidulants (hydrochloric acid, lactic acid and acetic acid) [67], [68], did not coincide with our microarray results on chitosan treatment. However, in the former study by Mols and co-workers [68], the gene encoding the Kdp ATPase A chain (BC0753) was significantly upregulated upon treatment with HCl pH 5.5 at growth suppressing conditions. The expression of BC0756 encoding the sensor protein KdpD was also upregulated due to non-lethal exposure to acetic acid in the same study. However, the significance of these findings to potassium acquisition is probably minor, as expression of other *kdp* genes or other genes related to potassium influx were not significantly altered. Finally, no altered expression of genes involved in potassium transport was described in different bacteria subjected to low-temperature stress, weak acid stress or low pH challenges, as reviewed by Beales [69].

In our study, chitosan treated *B. cereus* 14579 cells showed upregulation of genes encoding membrane proteins, whose expression was also found to be significantly altered in AS-48 or nisin treated cells of the same bacterium. Examples are the BC1612 (Na^+^/H^+^ antiporter) and BC4742 (permease) that were significantly upregulated in the presence of chitosan A and chitosan B (this study), AS-48 [65] and nisin (AT Kovacs and OP Kuipers, unpublished observations). However, the most upregulated operons in the two latter studies (i.e. the BC4206-BC4207 and the BC1453-BC1439 operons in the presence of AS-48 and nisin, respectively) were not affected by chitosan A or chitosan B.

Downregulation of a chitin-binding protein (BC2798) following exposure to both chitosan A and B might be a response to elevated levels of extracellular chitosan, which in structure only differs from chitin in fewer acetyl groups at the C-2 positions of the glucosamine units. Chitin-binding proteins are examples of carbohydrate-binding modules (CBM) that are present in many microorganisms utilizing chitin as a nutrient source. *B. subtilis* is among the species described to degrade shrimp shell waste, which contain chitin [70]. The function of CBMs is believed to be recognition and binding to chitin and thereby a synergistically action with chitinases to enhance the accessibility of the insoluble biopolymer chitin [71], [72]. In our study, however, expression of BC2798 was suppressed, not increased, as might be expected with increased substrate availability. In pathogenic bacteria, the CBMs have also recently been shown to be virulence factors involved in host tissue recognition [73]–[75]. The significance of our microarray data on chitin-binding protein is therefore not obvious.

In this study, we have provided further insight into bacterial response mechanisms to the biopolymer chitosan, and our findings coincide with the most feasible mode of action of chitosan, namely membrane permeabilization. The chitosans included were defined in their macromolecular properties, and in future experiments involving gene regulation upon exposure to this biopolymer, MW and F_A_ should be stated to elucidate if macromolecular characteristics are decisive of the bacterial response mechanisms. Also, the results obtained in this study should be compared with transcriptional responses to chitosan of various bacterial species possessing the Kdp system and also the Gram positive model organism *B. subtilis*, not containing any *kdp* genes, to see if potassium depletion caused by chitosan will activate transcription of genes involved in K^+^ transport.

## Materials and Methods

### Bacterial strain and culture conditions

*B. cereus* ATCC 14579 (the type-strain) was obtained from the American Type Culture Collection and is an enterotoxin-producing strain.

### Preparation of chitosans

Chitosans with F_A_ = 0.01 and 0.16 were obtained from FMC NovaMatrix (Sandvika, Norway). The samples were converted into water-soluble hydrochloride salts (chitosan-HCl) and partially depolymerized to obtain different DP ranges (DP  =  degree of polymerization  =  number of sugar residues per chain) as described elsewhere [76], [77]. Reduction of the degraded samples with NaBH_4_ was performed (reduction of terminal 2.5-dehydro-D-mannose), and average DP values were determined on basis of SEC-MALLS analysis, as previously described in Christensen et al. [78]. Data were processed and number and weight average molecular weights (M_n_ and M_w_, respectively) obtained as reported before [22].

Stock solutions of depolymerized chitosans of 4 mg/mL were prepared in Milli-Q grade water at 4°C overnight and adjusted to pH 4.0-4.5 before filtering (0.45 µm), aliquotation and storage at -20°C. An overview of the chitosans included in this study is found in Table 1.

### Microarray experiments

Exponentially growing cultures of the test strain inoculated from an overnight culture were grown in Iso-Sensitest Broth (Iso-SB) (Oxoid, Hampshire, England) containing 100 mM 4-Morpholineethanesulfonic acid (MES) (Sigma-Aldrich, St. Louis, MO) at pH 6 and 37°C, 225 rpm, to an optical density 2.5–3.0 at 600 nm (OD_600_) as measured with a Genesys 20 spectrophotometer (Thermo Fisher Scientific, Wilmington, US). A total of three independent biological replicates were included for both chitosan A and B. The average coefficient of variance values between the replicates were 53.3% and 32.6% for chitosan A and B, respectively. The maximum concentration of chitosan A or B not inhibiting growth, 50 µg/mL (final concentration), was added and cells harvested after 30 min by centrifugation (10.397× *g*, 1 min, RT). The pellets were immediately frozen in liquid nitrogen and stored at −80°C. RNA extraction was performed with the Macaloid/Roche protocol [79] with one additional step of phenol-chloroform washing. RNA concentration and purity was assessed using NanoDrop ND-1000 Spectrophotometer (Thermo Fisher Scientific). RNA samples were reverse transcribed into cDNA using the Superscript III reverse transcriptase kit (Invitrogen, Carlsbad, USA) and labelled with Cy3 or Cy5 monoreactive dye (GE Healthcare, Amersham, The Netherlands). Labelled and purified cDNA samples (Nucleospin Extract II, Biokè, Leiden, The Netherlands) were hybridized in Ambion Slidehyb #1 buffer (Ambion Europe Ltd) at 49°C for 18–20 h to DNA-microarrays containing amplicons of 5200 annotated genes from the genome of *B. cereus* 14579, where each open reading frame is represented by duplicates spots. The arrays were constructed as described elsewhere [80]. Slide spotting, slide treatment after spotting and slide quality control were done as before [81]. After hybridization, slides were washed for 5 min in 2x SSC with 0.5% SDS, 2 times 5 min in 1x SSC with 0.25% SDS, 5 min in 1x SSC 0.1% SDS, dried by centrifugation (2 min, 2.000 rpm) and scanned in GenePix 4200AL or GenePix 4000B Microarray Scanners (Axon Instruments, CA, US). Fluorescent signals were quantified using ArrayPro 4.5 (Media Cybernetics Inc., Silver Spring, MD, US) and further processed and normalized with MicroPrep [82]. CyberT [83] was used to perform statistical analysis. Genes with a Bayes P-value of ≤1.0×10^−4^ and ≥ twofold differentially expressed compared to the control, were considered significantly affected. Microarray data are MIAME compliant and the raw data have been deposited in a MIAME compliant Gene Expression Omnibus database (GSE29024), as detailed on the MGED Society website http://www.mged.org/Workgroups/MIAME/miame.html.

### Quantitative PCR

Nisin (0.5 µg/mL), bacitracin (25 µg/mL) and AS-48 (0.5 µg/mL) treated *B. cereus* 14579 samples were obtained as described earlier [65], while chitosan (50 µg/mL) challenged *B. cereus* samples were prepared as described above. At least 3 independent samples were included in the qPCR experiments. Following RNA purification (see above), samples were treated with RNase-free DNase I (Fermentas, St. Leon-Rot, Germany) for 60 min at 37 °C in DNaseI buffer (10 mM Tris·HCl (pH 7.5), 2.5 mM MgCl_2_, 0.1 mM CaCl_2_). Samples were purified with the Roche RNA Isolation Kit. Reverse transcription was performed with 50 pmol random nonamers on 4 µg of total RNA using RevertAid^TM^ H Minus M-MuLV Reverse Transcriptase (Fermentas). Quantification of cDNA was performed on a CFX96 Real-Time PCR System (BioRad, Hercules, CA) using Maxima SYBR Green qPCR Master Mix (Fermentas). The following primer sets were included in the experiments (Table 7): primer set 4 (BC0753) and primer set 5 (*rpoA* gene of *B. cereus*). The amount of BC0753 cDNA was normalized to the level of *rpoA* cDNA using the 2^−ΔΔCt^ method [84].

**Table 7:** Primers used in this study.^a^

Primer set number	Sequence (5′ to 3′)	
	Forward	Reverse
1	CGAATGCTGAGTGCAACAAG	GCTTTGCTACTAAAATAAAACGCGTCATTGTAAT
2	GGATCAAACGCGTTAAAATAGAGCCGACCTTTTTTGG	CTCCCAGAAACAAAGCCAAA
3	AGCGAGGCTCTATGAAACCA	GTTCAATCGGATGTGCCTTT
4	CAGCACATATTGAAGGGATGG	GCAATAGGAAGAAACACTCTCGT
5	CGTGGATATGGTACTACTTTGG	TTCTACTACGCCCTCAACTG

a Primer-incorporated *Mlu*I restriction sites are underlined.

### Construction of a *B. cereus* 14579 *kdp* deletion mutant

A Kdp ATPase/KdpD negative mutant was constructed by replacing the BC0753-BC0756 genes with the sequence ATGACGCGTTAA (5′-3′) using the markerless gene replacement method of Janes and Stibitz [85] with modifications. All PCRs were conducted in an Eppendorf Mastercycler gradient and DyNAzyme II DNA polymerase and dNTP Mix from Finnzymes (Finland) were used according to the instructions by the manufacturer. PCRs were performed using 95°C for 1 min, 30 cycles of 1 min at 95°C, 52°C for 1 min and 72°C for 1 min, before finally 72°C for 1 min, in an Eppendorf Mastercycler ep gradient S (Eppendorf AG, Hamburg, Germany). PCR products were analyzed by 1.0% agarose gel electrophoresis.

The upstream and downstream regions of the BC0753-BC0756 genes were amplified by PCR using genomic DNA from *B. cereus* 14579 and primer sets 1 and 2 (Table 7), respectively. The reverse primer of primer set 1 and forward primer of set 2 were modified to contain *Mlu*I restriction sites. Amplicons were cloned into pCR 2.1-TOPO (Invitrogen) and further transformed into *E. coli* One Shot TOP10 (Invitrogen). The downstream region (kdp down) was digested from the vector using *Mlu*I and *Xba*I and ligated into the *Mlu*I and *Xba*I sites of the pCR 2.1-TOPO containing the upstream region (kdp up). The complete construct (kdp up and kdp down) were now excised from pCR 2.1-TOPO using *Eco*RI and ligated into the corresponding restriction site of the thermosensitive shuttle vector pMAD [86] containing an additional I-Sce-I site. The pMADΔ*kdp* vector was introduced by electroporation into *B. cereus* 14579 electrocompetent cells, which were made essentially according to Mahillon et al. [87], but with the following modifications. The cultures were grown in BHI at 37°C, the centrifugation steps were carried out at room temperature and resuspension of the pellets after washing was done in 40% polyethylene glycol (PEG) 6000 (Merck, Darmstadt, Germany). Electroporation was performed in electroporation cuvettes (cat.no. 165–2086, Bio-Rad Laboratories, Hercules, CA) at 2.2 kV, 4 mS, with an Eppendorf Eporator apparatus (Eppendorf AG), and the cells were recovered in Luria-Bertoni broth (Oxoid) at 37°C, 150 rpm, for a minimum of 4 h. Integration of the vector plasmid (pMADΔ*kdp*) into the chromosome by recombination events (via homologous sequences) was performed as described by Arnaud et al. (2004) [86], and pBKJ233 containing the gene for the I-SceI enzyme was then introduced by electroporation, resulting in a double-stranded DNA break with subsequent repairing by homologous recombination and eventually the desired genetic replacement [85]. The deletion of the four genes was verified by PCR amplifications using oligonucleotides located upstream and downstream from the *kdp* operon (primer set 3, Table 7) on chromosomal DNA purified from clones. DNA sequencing was performed to confirm the construction of the *kdp* deletion mutant (Source BioScience Lifesciences, UK), and the sequence has been deposited in GenBank under accession number JN193502.

### Growth of *B. cereus* 14579 wild-type and *kdp* mutant strain in standard medium and under potassium limiting conditions

To compare growth of the *B. cereus* wild-type and mutant strain, these were grown in Iso-SB 100 mM MES at pH 6.0 for 9 h at 37°C, 160 rpm, inoculated from overnight cultures in the same medium, and plated onto blood agar plates to determine the growth yields. OD_600_ measurements were also performed in a Shimadzu UV-160A spectrophotometer (Shimadzu Corporation) to obtain growth curves for comparison. The potassium content of the growth medium supplemented with MES buffer was checked by the Central Laboratory at the Norwegian School of Veterinary Science (Oslo, Norway) using an ion selective electrode (Advia®1650, Siemens Medical Solutions Diagnostics) and determined to be 2.6 mM.

Growth of the two test strains in a modified Spizizen's minimal medium (SMM) [45] where no potassium was added, supplemented with 1.0 mg/mL arginine and a solution of trace elements [88], were compared. The potassium phosphates in SMM were replaced with equimolar amounts of the sodium salts. Final concentrations of 0, 1, 2 or 3 mM KCl were added to the medium. Overnight cultures of *B. cereus* 14579 (wild-type) and the *kdp* deletion strain were washed thrice in Spizizen's minimal salts [45] (potassium salts exchanged with sodium salts) and resuspended in the same salt solution after the final wash. Approximately 10^5^ CFU/mL of the test strains were added to SMM supplemented with arginine and trace elements and the cultures were incubated at 37°C, 160 rpm, for 18 h. Growth yields were determined by plating aliquots on blood agar plates at the end of the experiments, which were performed a minimum of three times. The presence of possible contaminating potassium in modified SMM supplemented with arginine and trace elements was not measured, but this is reported to be a maximum of 0.005% for the sodium salts and MgSO_4_×7H_2_0, according to the manufacturer (Merck, Darmstadt, Germany), which should not constitute more than 7 µM K^+^ altogether. This is below 20 µM K^+^, which was reported to be the contaminating amount of this ion in a minimal medium also containing sodium phosphates and applied in a study on K^+^ transport in *E. coli* cells [89]. The other chemicals included in our minimal medium (modified SMM and trace elements) are not declared to contain any contaminating K^+^.

### Osmotic upshift assay

Since the Kdp system is described to be important for coping with osmotic challenges and severe potassium limitations in *E. coli* [49], we decided to subject the *kdp* mutant strain and the wild-type strain to elevated concentrations of NaCl in an osmotic upshock assay. The two test strains grown in Iso-SB 100 mM MES at pH 6.0 until mid-log phase (OD_600_ 2.0–2.5) were exposed to final concentrations of 0.25, 0.5, or 1.0 M NaCl or water (control), and growth at 37°C, 160 rpm, were recorded as OD_600_ measurements (Shimadzu UV-160A) for 3 h after the addition of osmolytes. In *B. subtilis* subjected to osmotic upshock through addition of NaCl, the intracellular potassium level is described to increase to high values within the first hour [90]. The experiments were repeated at least three times.

### MIC and MBC determinations

*B. cereus* 14579 (wild-type) and the *kdp* mutant were included in a susceptibility assay with chitosan A and B (Table 1). Serial twofold dilutions of stock solutions of 4 mg/mL of the chitosans were prepared in MQ water in sterile 96-well flat-bottom microtiter plates (Becton Dickinson, France). Fresh cultures inoculated from overnight cultures of the test strains were grown in Iso-SB containing 100 mM MES at pH 6 and 37°C to an OD_600_ of 2.0–2.5 (mid-log phase). 100 µl volumes were added to each well in equal volume to the chitosan solution, yielding a bacterial test concentration of approximately 10^6^ CFU/mL. The microplates were incubated at 37°C for 20±1 h and MIC was read as the lowest concentration of chitosan inhibiting visible bacterial growth. MBC assays were performed by plating 100 µl aliquots from the wells onto blood agar plates and incubating at 37°C for 20 h. The MBC was defined as the lowest concentration reducing the inoculum by ≥99.9%.

## Supporting Information

Table S1 **Summary of transcriptional changes (Bayesian P≤1.0×10^-4^, cut-off value ≥2) in** ***B. cereus*** **14579 upon 50 µg/mL chitosan A treatment.**
(DOC)

Table S2 **Summary of transcriptional changes (Bayesian P≤1.0×10^-4^, cut-off value ≥2) in** ***B. cereus*** **14579 upon 50 µg/mL chitosan B treatment.**
(DOC)
