# Supplementary material for: Transcriptional Responses of Bacillus cereus towards Challenges with the Polysaccharide Chitosan
Source: PLoS One. 2011 Sep 8;6(9):e24304. doi: 10.1371/journal.pone.0024304 (PMC3169574; doi:10.1371/journal.pone.0024304)
Supplement: Table S1 — Summary of transcriptional changes (Bayesian P≤1.0×10^-4^, cut-off value ≥2) in B. cereus 14579 upon 50 µg/mL chitosan A treatment. (DOC) [file pone.0024304.s001.doc]

Table S1. Summary of transcriptional changes (Bayesian P ≤ 1.0 x 10^-4^, cut-off value ≥ 2) in *B. cereus* 14579 upon 50 μg/mL chitosan A treatment

| **locus tag** | **Expression ratio^a^** | **Significance (p-value)^b^** | **annotation^c^** | **feature^d^** |
| --- | --- | --- | --- | --- |
| **Upregulated** | | | | |
| **BC3719** | 8.7 | 10^-7^ | 1-phosphofructokinase | phosphomethylpyrimidine kinase domain |
| **BC0755** | 6.8 | 10^-5^ | potassium-transporting ATPase subunit C | SS, TMS(1) |
| **BC1043** | 6.7 | 10^-5^ | peptidylprolyl isomerase | SS, rotamase domain |
| **BC2609** | 5.9 | 10^-5^ | cytochrome P450 | p450 domain |
| **BC3720** | 5.8 | 10^-6^ | DeoR family transcriptional regulator | HTH |
| **BC4016** | 5.7 | 10^-6^ | cyclodextrin transport ATP-binding protein | AAA, transport-associated OB domain |
| **BC3718** | 5.1 | 10^-5^ | PTS system, fructose-specific II ABC component | phosphotransferase system domains |
| **BC0753** | 5.1 | 10^-4^ | potassium-transporting ATPase subunit A | TMS(10) |
| **BC2603** | 4.4 | 10^-4^ | hypothetical protein | SS, TMS(5) |
| **BC4366** | 4.3 | 10^-5^ | cystathionine beta-lyase |  |
| **BC4015** | 4.1 | 10^-5^ | oligo-1,6-glucosidase | amylase domain |
| **BC0754** | 3.7 | 10^-5^ | potassium-transporting ATPase subunit B | TMS(3), AAA, hydrolase |
| **BC4062** | 3.7 | 10^-5^ | hypothetical protein | SS, CD |
| **BC3515** | 3.6 | 10^-4^ | glycosyltransferase |  |
| **BC4761** | 3.6 | 10^-4^ | methionine adenosyltransferase | S-adenosylmethionine synthetase domains |
| **BC5448** | 3.6 | 10^-4^ | UDP-glucose 4-epimerase | epimerase |
| **BC3466** | 3.5 | 10^-5^ | ferrichrome-binding protein | SS, PPD |
| **BC1461** | 3.5 | 10^-5^ | DNA integration/recombination/invertion protein | integrase domain |
| **BC4242** | 3.5 | 10^-5^ | H^+^/Na^+^-glutamate symport protein | SS, Na^+^:dicarboxylate symporter domain |
| **BC4802** | 3.4 | 10^-5^ | hypothetical protein | SS |
| **BC5387** | 3.4 | 10^-4^ | phosphotransacetylase |  |
| **BC0413** | 3.4 | 10^-5^ | exo-α-1,4-glucosidase | amylase domain |
| **BC5380** | 3.3 | 10^-4^ | ferrichrome-binding protein | SS, PPD |
| **BC3423** | 3.3 | 10^-5^ | ArsR family transcriptional regulator | HTH |
| **BC1528** | 3.2 | 10^-5^ | hypothetical protein | TMS(4), peptidase |
| **BC3523** | 3.1 | 10^-5^ | hemolysin II | leukocidin domain |
| **BC2969** | 3.0 | 10^-4^ | hypothetical protein | monooxygenase domain |
| **BC4742** | 2.9 | 10^-4^ | ABC transporter permease protein | SS, TMS(4), FtsX |
| **BC0660** | 2.9 | 10^-5^ | ribokinase |  |
| **BC4813** | 2.8 | 10^-5^ | hypothetical protein |  |
| **BC2772** | 2.8 | 10^-5^ | methyltransferase | methyltransferase |
| **BC4831** | 2.7 | 10^-5^ | ABC transporter ATP-binding protein | AAA |
| **BC5370** | 2.7 | 10^-5^ | agmatinase |  |
| **BC2214** | 2.7 | 10^-4^ | small heat shock protein | heat shock protein domain |
| **BC4209** | 2.7 | 10^-4^ | lipoate-protein ligase A | lipoate-protein ligase domain |
| **BC1612** | 2.6 | 10^-4^ | Na^+^/H^+^ antiporter NapA (inosine-dependent germination) | TMF(11) |
| **BC1355** | 2.6 | 10^-4^ | ribonucleotide-diphosphate reductase subunit beta | ribonuclease domain |
| **BC3070** | 2.6 | 10^-4^ | signal peptidase I | SS, TMS(1), peptidase domain |
| **BC4248** | 2.6 | 10^-4^ | endonuclease IV | TMS(1) |
| **BC0483** | 2.5 | 10^-4^ | penicillin-binding protein | TMS(1), β-lactamase domain |
| **BC3738** | 2.5 | 10^-4^ | Fe(III) dicitrate-binding protein | SS, PPD |
| **BC1828** | 2.5 | 10^-4^ | Xaa-Pro aminopeptidase | peptidase domain |
| **BC4119** | 2.4 | 10^-4^ | adenosylmethionine-8-amino-7-oxononanoate transaminase | aminotransferase domain |
| **BC3418** | 2.3 | 10^-4^ | phenazine biosynthesis protein |  |
| **BC3541** | 2.3 | 10^-4^ | flavodoxin |  |
| **BC4367** | 2.3 | 10^-4^ | cysteine synthase | pyrodoxal phosphate binding domain |
| **BC1030** | 2.3 | 10^-4^ | hypothetical protein | SS |
| **BC4017** | 2.3 | 10^-4^ | hypothetical protein | short-chain dehydrogenase domain |
| **BC4981** | 2.3 | 10^-4^ | cysteine desulfhydrase | aminotransferase domain |
| **BC4528** | 2.3 | 10^-4^ | ferrichrome-binding protein | SS, PPD |
| **BC4496** | 2.2 | 10^-4^ | glutamate racemase | racemase domain |
| **BC1168** | 2.1 | 10^-4^ | ClpB protein | AAA(2) |
| **BC4373** | 2.1 | 10^-4^ | hypothetical protein | SS |
| **BC0464** | 2.1 | 10^-4^ | thioredoxin-like oxidoreductase | radical SAM domain |
| **BC0662** | 2.1 | 10^-4^ | ribose ABC transporter ATP-binding protein | AAA(2) |
| **BC3958** | 2.1 | 10^-4^ | myo-inositol-1(or 4)-monophosphatase |  |
| **BC1035** | 2.1 | 10^-4^ | glycerol kinase |  |
| **Downregulated** | | | | |
| **BC2292** | 0.5 | 10^-4^ | 3-hydroxyisobutyryl-CoA hydrolase | crotonase domain |
| **BC0806** | 0.5 | 10^-4^ | BigG family transcription antiterminator | HTH(2), PTS (phosphotransferase system) regulator domain |
| **BC4148** | 0.5 | 10^-4^ | arginine ABC transporter ATP-binding protein | AAA |
| **BC3698** | 0.5 | 10^-4^ | cell wall endopeptidase | peptidase domain |
| **BC2289** | 0.5 | 10^-4^ | 3-hydroxyisobutyrate dehydrogenase | NAD binding domain |
| **BC2991** | 0.5 | 10^-4^ | uridine kinase |  |
| **BC4086** | 0.5 | 10^-4^ | purine nucleoside phosphorylase | phosphorylase domain |
| **BC0745** | 0.5 | 10^-4^ | hydroxymethylpyrimidine-binding protein | SS |
| **BC0742** | 0.4 | 10^-4^ | transcriptional activator *tenA* |  |
| **BC2565** | 0.4 | 10^-4^ | Phage protein |  |
| **BC4630** | 0.4 | 10^-4^ | argininosuccinate synthase |  |
| **BC1483** | 0.4 | 10^-4^ | ferredoxin |  |
| **BC1822** | 0.4 | 10^-4^ | pyrimidine-nucleoside phosphorylase |  |
| **BC0410** | 0.4 | 10^-4^ | Crp family transcriptional regulator | cNMP binding domain, HTH |
| **BC5314** | 0.4 | 10^-4^ | hypothetical protein |  |
| **BC0743** | 0.4 | 10^-5^ | hydroxymethylpyrimidine transport ATP-binding protein | AAA |
| **BC2119** | 0.4 | 10^-4^ | respiratory nitrate reductase beta chain | oxidoreductase domain |
| **BC0378** | 0.4 | 10^-4^ | 5-methylribose kinase |  |
| **BC2128** | 0.4 | 10^-5^ | nitrite extrusion protein | SS, TMS(11) |
| **BC1823** | 0.4 | 10^-5^ | cytidine deaminase |  |
| **BC3222** | 0.4 | 10^-4^ | ABC transporter ATP-binding protein | AAA |
| **BC4149** | 0.4 | 10^-4^ | arginine ABC transporter permease | TMS(3) |
| **BC1821** | 0.4 | 10^-4^ | nucleoside permease *nupC* | SS, TMS(8) |
| **BC2134** | 0.3 | 10^-4^ | bifunctional uroporphyrinogen-III | methylase domain |
| **BC0744** | 0.3 | 10^-5^ | hydroxymethylpyrimidine transport system permease protein | SS, TMS(6) |
| **BC4927** | 0.3 | 10^-5^ | cell surface protein | TMS(2) |
| **BC3855** | 0.3 | 10^-4^ | putative alkaline-shock protein |  |
| **BC2121** | 0.3 | 10^-5^ | respiratory nitrate reductase γ chain | nitrate reductase domain |
| **BC3223** | 0.3 | 10^-6^ | ABC transporter permease protein | SS, FtsX |
| **BC0492** | 0.3 | 10^-5^ | pyruvate formate-lyase activating enzyme | radical SAM domain |
| **BC0402** | 0.3 | 10^-5^ | cystine-binding protein | SS, bacterial periplasmic substrate-binding proteins |
| **BC3651** | 0.3 | 10^-6^ | urocanate hydratase | urocanase |
| **BC0403** | 0.3 | 10^-5^ | glutamine transport ATP-binding protein glnQ | AAA |
| **BC2778** | 0.3 | 10^-4^ | acetoin dehydrogenase E1 component β-subunit | transketolase |
| **BC0404** | 0.3 | 10^-5^ | methyl-accepting chemotaxis protein | SS, TMS(2), histidine kinases/adenylyl cyclases/methyl binding proteins/phosphatases domain |
| **BC2132** | 0.3 | 10^-6^ | precorrin-2 dehydrogenase |  |
| **BC4793** | 0.3 | 10^-5^ | cytochrome d ubiquinol oxidase, subunit II | cytochrome oxidase domain |
| **BC2798** | 0.2 | 10^-4^ | chitin binding protein | chitin binding domain, carbohydrate-binding domain |
| **BC2133** | 0.2 | 10^-6^ | CbiX protein | CbiX domains |
| **BC2136** | 0.2 | 10^-6^ | nitrite reductase [NAD(P)H] large subunit | oxidoreducate, ferredoxin domain |
| **BC0503** | 0.2 | 10^-5^ | hypothetical protein | SS, CD, TMS(2) |
| **BC2779** | 0.2 | 10^-4^ | acetoin dehydrogenase E1 component α-subunit | dehydrogenase |
| **BC0412** | 0.2 | 10^-4^ | FAD-dependent oxidase | FAD-binding domain |
| **BC3650** | 0.2 | 10^-6^ | imidazolonepropionase | amidohydrolase |
| **BC2776** | 0.2 | 10^-7^ | dihydrolipoamide dehydrogenase | oxidoreductasedomain |
| **BC2777** | 0.2 | 10^-7^ | branched-chain alpha-keto acid dehydrogenase subunit E2 | biotin attachment domain, dehydrogenase domain |
| **BC0406** | 0.1 | 10^-6^ | arginine deiminase | aminidotransferase |
| **BC0407** | 0.1 | 10^-7^ | ornithine carbamoyltransferase | carbamoyl-P binding domain; Asp/Orn binding domain |
| **BC0409** | 0.1 | 10^-7^ | carbamate kinase | kinase |
| **BC2992** | <0.1 | 10^-9^ | ribosomal-protein-alanine acetyltransferase | acetyltransferase |
| **BC0408** | <0.1 | 10^-8^ | arginine/ornithine antiporter | permease; TMS(1) |
| ^a^ The ratio of gene expression is shown. Ratio: expression in chitosan treated samples over that in untreated samples.  ^b^ Bayesian *p* value  ^c^ Putative function of protein as annotated in the *B. cereus* ATCC14579 genome sequence  ^d^ Domains detected using SMART search (http://smart.embl-heidelberg.de/) [40]. SS, signal sequence; TMS(n), transmembrane segment (n is the number of such domain); CD, conserved domain of unknown function; PPD, periplasmic domain; HTH, helix turn helix,; FtsX, FtsX like permease family; AAA, ATPase domain. | | | | |
