# Supplementary material for: Transcriptional Responses of Bacillus cereus towards Challenges with the Polysaccharide Chitosan
Source: PLoS One. 2011 Sep 8;6(9):e24304. doi: 10.1371/journal.pone.0024304 (PMC3169574; doi:10.1371/journal.pone.0024304)
Supplement: Table S2 — Summary of transcriptional changes (Bayesian P≤1.0×10^-4^, cut-off value ≥2) in B. cereus 14579 upon 50 µg/mL chitosan B treatment. (DOC) [file pone.0024304.s002.doc]

Table S2. Summary of transcriptional changes (Bayesian P ≤ 1.0 x 10^-4^, cut-off value ≥ 2) in *B. cereus* 14579 upon 50 μg/mL chitosan B treatment

| **locus tag** | **Expression ratio^a^** | **Significance (p-value)^b^** | **annotation^c^** | **feature^d^** |
| --- | --- | --- | --- | --- |
| **Upregulated** | | | | |
| **BC0753** | 7.5 | 10^-8^ | Potassium-transporting ATPase A chain | TMS(10) |
| **BC0754** | 6.9 | 10^-7^ | Potassium-transporting ATPase B chain | TMS(3), AAA, hydrolase |
| **BC0755** | 6.4 | 10^-9^ | Potassium-transporting ATPase C chain | SS, TMS(1) |
| **BC0814** | 4.3 | 10^-5^ | ABC transporter permease protein | TMS(1), FtsX |
| **BC1739** | 3.5 | 10^-4^ | H^+^/Na^+^-glutamate symport protein | TMS(9) |
| **BC1461** | 3.3 | 10^-5^ | DNA integration/recombination/invertion protein | integrase |
| **BC4813** | 3.3 | 10^-5^ | hypothetical protein |  |
| **BC0756** | 3.3 | 10^-6^ | sensor protein (KdpD) | universal stress protein domain |
| **BC3738** | 3.2 | 10^-5^ | Iron(III) dicitrate-binding protein | SS, PPD |
| **BC1612** | 3.1 | 10^-6^ | Na^+^/H^+^ antiporter NapA (inosine-dependent germination) | TMS(11) |
| **BC3093** | 3.1 | 10^-4^ | aspartate ammonia-lyase | lyase, fumarase |
| **BC5448** | 3.0 | 10^-4^ | UDP-glucose 4-epimerase | epimerase |
| **BC0816** | 3.0 | 10^-5^ | periplasmic component of efflux system | SS, superfamily of outer membrane efflux proteins |
| **BC1542** | 2.8 | 10^-4^ | aspartate alpha-decarboxylase | decarboxylase domain |
| **BC0815** | 2.7 | 10^-6^ | ABC transporter ATP-binding protein | AAA |
| **BC4742** | 2.6 | 10^-5^ | ABC transporter permease protein | SS, TMS(9) |
| **BC4831** | 2.6 | 10^-4^ | ABC transporter ATP-binding protein | AAA |
| **BC4802** | 2.5 | 10^-5^ | hypothetical protein | SS |
| **BC2214** | 2.5 | 10^-5^ | small heat shock protein | heat shock protein domain |
| **BC2603** | 2.5 | 10^-4^ | hypothetical protein | SS, TMS(5) |
| **BC4583** | 2.4 | 10^-4^ | glyceraldehyde-3-phosphate dehydrogenase |  |
| **BC4761** | 2.3 | 10^-4^ | methionine adenosyltransferase | S-adenosylmethionine synthetase domains |
| **BC4830** | 2.2 | 10^-4^ | ABC transporter permease protein | TMS(4), FtsX |
| **BC0757** | 2.2 | 10^-6^ | bicyclomycin resistance protein | SS, TMS(12) |
| **BC4269** | 2.2 | 10^-5^ | phosphate-binding protein |  |
| **BC1355** | 2.1 | 10^-4^ | ribonucleotide-diphosphate reductase subunit beta | ribonuclease domain |
| **BC0817** | 2.1 | 10^-5^ | hypothetical protein | TMS(7) |
| **BC0900** | 2.1 | 10^-4^ | hypothetical protein | TMS(2) |
| **BC1354** | 2.0 | 10^-5^ | ribonucleotide-diphosphate reductase subunit alpha |  |
| **BC0712** | 2.0 | 10^-4^ | phosphate ABC transporter permease | SS, TMS(6) |
| **Downregulated** | | | | |
| **BC2135** | 0.5 | 10^-5^ | nitrite reductase [NAD(P)H] small subunit |  |
| **BC0411** | 0.5 | 10^-4^ | hypothetical protein | TMS(2) |
| **BC5285** | 0.5 | 10^-4^ | bacitracin transport ATP-binding protein | AAA |
| **BC2118** | 0.5 | 10^-4^ | respiratory nitrate reductase alpha chain | molybdopterin oxidoreductase Fe_4_S_4_ domain |
| **BC2056** | 0.5 | 10^-4^ | hypothetical protein | SS, TMS(2) |
| **BC0410** | 0.5 | 10^-4^ | Crp family transcriptional regulator | cNMP binding domain, HTH |
| **BC4240** | 0.5 | 10^-4^ | transcriptional regulator |  |
| **BC2802** | 0.5 | 10^-6^ | hypothetical protein | SS, TMS(3) |
| **BC2136** | 0.5 | 10^-5^ | nitrite reductase [NAD(P)H] large subunit |  |
| **BC4025** | 0.4 | 10^-5^ | hypothetical protein |  |
| **BC3651** | 0.4 | 10^-4^ | urocanate hydratase |  |
| **BC0503** | 0.4 | 10^-4^ | hypothetical protein | SS, TMS(4) |
| **BC3650** | 0.4 | 10^-5^ | imidazolonepropionase |  |
| **BC2220** | 0.4 | 10^-5^ | alcohol dehydrogenase |  |
| **BC0378** | 0.4 | 10^-4^ | 5-methylribose kinase |  |
| **BC0194** | 0.4 | 10^-5^ | hypothetical protein | SS, TMS(2) |
| **BC4548** | 0.4 | 10^-5^ | cell surface protein | SS, near transporter domain |
| **BC3652** | 0.4 | 10^-4^ | histidine ammonia-lyase |  |
| **BC2134** | 0.4 | 10^-5^ | methyltransferase/uroporphyrinogen-III synthase |  |
| **BC3222** | 0.4 | 10^-4^ | ABC transporter ATP-binding protein | AAA |
| **BC5141** | 0.4 | 10^-4^ | central glycolytic genes regulator |  |
| **BC4792** | 0.4 | 10^-4^ | cytochrome d ubiquinol oxidase subunit I | SS, TMS(8) |
| **BC0492** | 0.4 | 10^-6^ | pyruvate formate-lyase activating enzyme |  |
| **BC2798** | 0.2 | 10^-5^ | chitin binding protein | chitin binding domain, carbohydrate-binding domain |
| ^a^ The ratio of gene expression is shown. Ratio: expression in chitosan treated samples over that in untreated samples.  ^b^ Bayesian *p* value  ^c^ Putative function of protein as annotated in the *B. cereus* ATCC14579 genome sequence  ^d^ Domains detected using SMART search (http://smart.embl-heidelberg.de/) [40] SS, signal sequence; TMS(n), transmembrane segment (n is the number of such domain); FtsX, FtsX like permease family; AAA, ATPase domain; PPD, periplasmic domain. | | | | |
